# Chronic urticaria treatment challenges in children

**DOI:** 10.1590/1984-0462/2025/43/2024107

**Published:** 2024-12-20

**Authors:** Luis Felipe Ensina, Larissa Silva Brandão, Ana Caroline Dela Bianca Melo, Martti Antila, Moshe Ben-Shoshan, Dirceu Solé

**Affiliations:** a Universidade Federal de São Paulo, São Paulo, SP, Brazil.; b Universidade Federal de Pernambuco, Recife, PE, Brazil.; c Pontíficia Universidade Católica de São Paulo, Sorocaba, (SP), Brazil.; d McGuill University, Montreal Children’s Hospital, Montreal, Canada.

**Keywords:** Chronic urticaria, Children, Treatment, Urticária crônica, Crianças, Tratamento

## Abstract

**Objective:**

This paper aims to review the efficacy and safety of current chronic urticaria (CU) treatment in children and the existing patient-reported outcome measures (PROMs) used in this age group.

**Data source:**

Since there are few studies of CU in children, the authors performed a non-systematic review of published articles in English, Spanish, and Portuguese in the PubMed database in the last decade. Keywords used were (antihistamines OR omalizumab OR cyclosporine OR treatment) AND (chronic urticaria) AND (children OR adolescents).

**Data synthesis:**

According to the current guideline’s algorithm, the treatment of CU involves using high doses of antihistamines when there is no response with the licensed dosage. The effectiveness of this increase in children has been demonstrated with control rates ranging from 35% to 92%, with few studies addressing safety profiles. Omalizumab is approved for children over 12 years of age as a second step in the algorithm. Although more studies with children are needed to assess its effectiveness and safety, some data show complete control of symptoms in more than 80% of pediatric cases with no adverse effects, including in children under 12 years. When monitored closely, cyclosporine showed a good response rate in pediatric CU with few adverse events. Also, PROMs validated for this age can be helpful in clinical decisions, such as Urticaria Activity Score summed over 7 days, Urticaria Control Test, and Chronic Urticaria Quality of Life Questionnaire.

**Conclusions:**

Collaborative studies are necessary to generate stronger evidence to support the guideline recommendations for children. The existing data indicate that these drugs are safe and effective for treatment when dose adjustments are made.

## RESUMO

**Objetivo:**

Esta revisão avalia o tratamento da urticária crônica (UC), sua eficácia e segurança em crianças, além das ferramentas de avaliação de resultados (PROMS) específicas para essa faixa etária.

**Fonte de dados:**

Dada a escassez de estudos em crianças, os autores realizaram uma revisão não sistemática de artigos publicados em inglês, espanhol e português na última década nos bancos de dados da United States National Library of Medicine (PubMed), Scientific Electronic Library Online (SciELO) e Embase. As palavras-chave utilizadas foram anti-histamínicos OU omalizumabe OU ciclosporina OU tratamento E urticária crônica E crianças OU adolescentes.

**Síntese dos dados:**

O tratamento padrão da UC envolve o uso de altas doses de anti-histamínicos em casos refratários à dose licenciada. Estudos mostraram eficácia desse aumento de dose em crianças, com taxas de controle variando entre 35 e 92%, embora haja poucos estudos sobre segurança. O omalizumabe é aprovado para crianças com mais de 12 anos de idade como segunda etapa de tratamento, com alguns dados indicando controle completo dos sintomas em mais de 80% dos casos infantis, sem eventos adversos, mesmo em menores de 12 anos. A ciclosporina, quando monitorada adequadamente, mostrou boa resposta na UC pediátrica, com poucos eventos adversos relatados. Além disso, PROMS validados para a idade, como UAS7, UCT, CU-Q2oL, podem ser úteis nas decisões clínicas.

**Conclusões:**

Embora mais estudos colaborativos sejam necessários para apoiar as recomendações das diretrizes em crianças, os dados existentes demonstram que esses medicamentos são seguros e eficazes para o tratamento da UC pediátrica, desde que haja ajuste adequado de doses.

## INTRODUCTION

Urticaria is a condition characterized by the development of wheals (hives), angioedema, or both. It is classified based on its duration, as acute or chronic (>6 weeks), and the role of definite triggers, as inducible or spontaneous.^1^

Chronic urticaria (CU) is estimated to be present in 0.5–5% of the general population.^2^ In children, the point prevalence is 1.43%, and it is similar in male and female children aged less than 15 years.^3^ Angioedema is reported in 5–50% of cases of children with CU.^4,5^ Although data on the natural history of pediatric CU are still scarce, in previous studies, the median age of CU onset was 5–9 years.^6^

The resolution rate of CU in children is low, with only 10.3 per 100 patients per year achieving complete control of symptoms. The most common subtype is chronic spontaneous urticaria (CSU), although chronic inducible urticaria (CIndU) may affect up to 20% of children with CU.^7^ The resolution rate is reported to be even lower in inducible forms, for example, cold-induced urticaria in children is reported to have a 1-year resolution rate of approximately 5% (4.8 cases of disease resolution per 100 patient-years) versus 10% for CSU.^8^

The impact of CU on a patient’s quality of life can be significant, with pruritus and swelling causing sleep disturbances and affecting daily activities. Therefore, it is crucial to establish a correct diagnosis to initiate appropriate therapies.

The goal of urticaria treatment is to completely eliminate symptoms and normalize patient’s quality of life. Patient-reported outcome measures (PROMs) have been successfully used to support clinical decision and management strategies in patients with CU. The current international guideline for diagnosis and treatment of urticaria is primarily focused on adult-based studies and recommends a three-step treatment algorithm starting with non-sedating H1-antihistamines, followed by add-on omalizumab for H1-histamines non-responders, and ciclosporin when omalizumab is not available or fails to control the disease. The management of urticaria in children is often extrapolated from these recommendations, and this can pose a challenge for allergists and dermatologists when treating pediatric CU.^1^

In this paper, we aim to review the efficacy and safety of current CU treatment in children and the existing PROMs used in this age group to access disease activity, control, and quality of life.

## METHOD

Since there is a lack of randomized controlled trials in children for most of the recommended CU treatments, the authors performed a non-systematic review of published articles in English, Spanish, and Portuguese in the PubMed database in the last decade, from 2014 to 2024. Keywords used were (antihistamines OR omalizumab OR cyclosporine OR treatment) AND (chronic urticaria) AND (children OR adolescents), yielding 573 results. Additional searches were conducted based on an initial literature review and the authors’ expertise, with further selection based on relevance from other databases such as SciELO, EMBASE, and SCOPUS.

## RESULTS

### Evaluating disease activity and control in children

Evaluating CU activity and its control is fundamental for monitoring and making the best therapeutic decisions for each patient. PROMs in urticaria have already been well-established for adults. Initially developed for research purposes in clinical trials, PROMs have been increasingly recognized as fundamental and indispensable tools in medical diagnosis and treatment from the patient’s perspective. Currently, these instruments are available in a wide range of languages to assess disease activity (Urticaria Activity Score summed over 7 days [UAS7] and Angioedema Activity Score [AAS]), quality of life (Chronic Urticaria Quality of Life Questionnaire [CU-Q2oL] and Angioedema Quality of Life Questionnaire [AE-QoL]), and control (Urticaria Control Test [UCT] and Angioedema Control Test [AECT]).^1^ Although these tools have been validated in adults, older children and adolescents usually have no difficulties understanding and using them (Table 1).

**Table 1:** Patient-reported outcome measures (PROMs) available for assessing activity, control, and quality of life in patients with urticaria and/or angioedema.

	Urticaria	Angioedema
Activity	UAS7	AAS
Quality of life	CU-Q2oL	AE-QoL
Disease control	UCT	UCT/AECT

UAS7: 7-day Urticaria Activity Score; AAS: Angioedema Activity Score; CU-Q2oL: Urticaria Quality of Life Questionnaire; AE-QoL: Angioedema Quality of Life Questionnaire; UCT: Urticaria Control Test.

**Figure 1:**
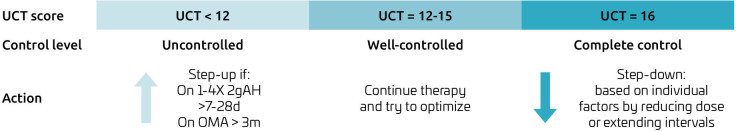
Chronic urticaria: management decisions and treatment adjustments.^1^

CIndU: chronic inducible urticaria; d: days; m: months; OMA: omalizumab; 2gAH: second-generation H1-antihistamine; UCT: Urticaria Control Test.

The UCT is a simple four-question retrospective tool that can be used in CSU and CIndU to assess disease control, even in patients who present isolated angioedema. It is the international guideline-recommended tool to step up or step down in the treatment algorithm (Figure 1).^1^ A study of 52 children with CU (35 with CSU) has demonstrated that the same UCT questionnaire used for adults can also be applied to children, representing an important step in the right direction for treating CU.^9^

Another study with a small sample suggests that the UAS7 is a useful prospective tool to evaluate disease activity in children with CSU, as it correlates with children’s quality-of-life assessments and treatment effectiveness, resulting in a decrease in UAS7 scores over 7 days. However, further large-scale studies are needed to validate the use of UAS7 in children due to the limited sample size.^10^

Alternative tools validated in the pediatric population include the Children’s Dermatology Life Quality Index (CDLQI), which is not specific to urticaria but to dermatological diseases, and the Pediatric Itch Severity Scale.^11,12^ Several studies agree that CU is a disabling skin disease that significantly impacts the patient’s psychological state and quality of life. In a mixed population of children and adults, patients with an uncertain diagnosis were found to have higher levels of anxiety and depression.^13^ The CDLQI is the most commonly used tool for assessing children’s quality of life;^14^ however, CU-Q2oL has been recommended for CU patients.^1^ Unfortunately, most studies on psychological aspects and the impact on quality of life have focused on adult patients, with limited research on children. Pediatric disease can significantly influence the parent-child relationship, becoming central in family life. Parents may experience guilt, frustration, and financial strain, while children may perceive themselves as very ill and develop a personal identity centered around the disease.^15^ In this context, evaluating the family’s characteristics, resources, and social context becomes crucial to prevent possible experiences of limitation and fragility in the child.

Thus, developing and validating PROMs for the pediatric population will address a significant gap and prove valuable for both clinical practice and research.^1^

### Antihistamines

**Figure 2:**
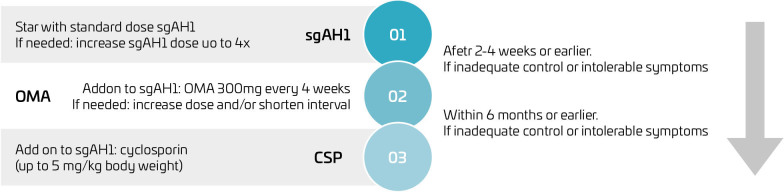
Recommended treatment algorithm for chronic urticaria.^1^

It is widely agreed that caution should be exercised when applying the same adult treatment algorithm for CU to the pediatric age group, considering safety, efficacy, and the approved age for each medication. According to this algorithm, the recommended treatment involves using second-generation H1 antihistamines (sgAH) daily (Figure 2). If symptoms are not adequately controlled within 2–4 weeks, the dosage should be increased to four times the approved dose (Table 2).^1^

sgAH1: second-generation H1-antihistamines; OMA: omalizumab; CSP: cyclosporin.

**Table 2:** Second-generation antihistamines available for use in the pediatric population.

H1 Antihistamines	Age approved (FDA/EMA)	Presentation (in Brazil)^49^	Dosage^49^
Cetirizine	>2 years	Drops (10 mg/mL) Oral solution (1 mg/mL)	2–6 years: 5 drops bid or 10 drops qd. 6–12 years: 10 drops bid >12 years: 20 drops qd 2–6 years: 2.5 mL bid 6–12 years: 5 mL bid or 10 mL qd >12 years: 10 mL qd
Desloratadine	>6 months	Drops (1.25 mg/mL) Oral solution (0.5 mg/mL) Tablets (5 mg)	6–11 m: 2 mL qd 1–5 years: 20 drops qd 6–11 years: 40 drops qd >12 years: 80 drops qd 6–11 m: 2 mL qd 1–5 years: 2.5 mL qd 6–11 years: 5 mL qd >12 years: 10 mL qd >12 years: 1 tablet qd
Ebastine	>2 years	Oral solution (1 mg/mL) Tablets (10 mg)	2–5 years: 2.5 mL qd 6–11 years: 5 mL qd >12 years: 1 tablet qd
Epinastine	>6 years	Syrup (10 mg/5 mL) Tablets (10 mg, 20 mg)	6–11 years: 2.5–5 mL qd >12 years: 1 tablet (10 mg or 20 mg) qd
Fexofenadine	>6 months	Oral suspension (6 mg/mL) Tablets (60 mg, 120 mg, 180 mg)	6 m–23 m: 2.5 mL bid (30 mg/day) 2–11 years: 5 mL bid (60 mg/day) >12 years: 1 tablet 180 mg qd
Levocetirizine	>2 years	Drops (5 mg/mL)	2–6 years: 5 drops bid >6 years: 20 drops qd
Loratadine	>2 years	Syrup (1 mg/mL) Tablets (10 mg)	2–12 years: <30 kg 5 mL qd >30 kg 10 mL qd >12 years: 10 mL qd >12 years: 1 tablet qd
Rupatadine	>12 years*	Tablets (10 mg)	>12 years: 1 tablet qd
Bilastine	>6 years*	Oral solution (2.5 mg/mL) Tablets (20 mg)	6–11 years: 4 mL qd >12 years: 8 mL qd >12 years: 1 tablet qd

*The minimum licensed age may vary depending on the country and specific formulation (syrup, tablets, chewable tablets); for example, in Canada, rupatadine is approved for 2 years and older and bilastine for 4 years and older.

FDA: Federal Drug Administration; EMA: European Medicines Agency; bid: twice a day; qd: once a day.

The effectiveness of these dosage increases has been demonstrated in the pediatric age group, with control rates ranging from 35 to 92% of patients.^16-18^ Balp et al. reported that 35.7% of 98 children with CSU required higher doses of H1-antihistamines to control the disease, while 11.4% were refractory to them. They also observed that most patients requiring high doses were between 2 and 6 years of age. However, no data on high dose safety were reported.^18^ Similarly, a study comparing children and adults with CSU revealed that response to antihistamines was higher in the pediatric group, and only 7% used omalizumab versus 20.8% in adults.^4^

The GA(2)LEN position paper strongly advises against the use of first-generation H1-antihistamines in both adults and children.^19^ This view is shared by the WHO guideline on Allergic Rhinitis and its Impact on Asthma,^20^ as this generation of antihistamines poorly selects the H1 receptor and can cross the blood-brain barrier, impacting sleep during the REM phase, school learning, and may cause paradoxical excitability, constipation, and weight gain.^1,21,22^

There are several options of second-generation H1-antihistamines with proven efficacy and safety in the pediatric population in licensed dosages (Table 2).^23,24^ The choice of which sgAHs to use in children with urticaria should consider factors such as age and availability, as not all of them are available in syrup or fast-dissolving tablet forms suitable for children. The approved minimum age for use also varies from country to country. All subsequent steps should be based on individual considerations and taken with care, such as the up-dosing of antihistamines.^1^

Few randomized controlled trials (RCTs) assessed the efficacy and safety of up-dosing sgAHs for pediatric CSU management. Two double-blind RCTs, including children over 12 years of age, demonstrated similar efficacy of double-dosing rupatadine compared to licensed dosage.^25,26^ Another RCT tested fexofenadine up to four-fold the standard dose against placebo in adolescents. The improvement of urticaria symptoms with increasing doses of fexofenadine followed a linear trend, at which point efficacy plateaued.^27^ Regarding safety, all studies reported similar profiles in all treatment dosage groups, except for the rupatadine double-dose group that had a slightly increased incidence of adverse events, most commonly headache and somnolence.^26^

More studies are still needed to establish the safety of sgAH up-dosing recommendations in children, as there are for adults. However, following the up-dosing recommendations is advised and should be done with good judgment.

### Omalizumab

Omalizumab is a recombinant humanized anti-IgE monoclonal antibody approved for the treatment of CSU in children aged 12 years and older, as well as adults. It is recommended as a second-line treatment for adults and adolescents who are refractory to high-dose antihistamines.^1^ However, there are limited data available regarding its efficacy and safety in the pediatric population.

Phase III clinical trials of omalizumab for CSU (ASTERIA I and II, and GLACIAL) included 39 adolescents aged 12 to under 18 years (4% of the total study population). In these trials, patients were required to be refractory to standard doses of H1-antihistamines in ASTERIA I and II and up to four-fold the approved dose in GLACIAL, along with H2-antihistamines, leukotriene antagonists, or both. Although some differences between the adult and adolescent populations were described in the studies, specific data regarding efficacy and safety in patients under 18 years of age were not published.^28^

A systematic review of the literature included 14 children under 12 years of age (median age 9.5 years, range 2–11 years) treated with omalizumab for various types of urticaria, including CSU, solar urticaria, and cold-induced urticaria. All patients were refractory to high-dose H1-antihistamines or combination therapy (H2-histamine blockers, leukotriene receptor antagonists), and some had been previously treated with cyclosporine or oral corticosteroids. Omalizumab doses in this age group varied from 75 to 300 mg administered monthly, with lower doses often sufficient for symptom control in children less than 6 years of age. Overall, 81% of patients with CSU achieved complete control of symptoms, and 19% achieved partial control, with a median time to response of 4 weeks. No significant adverse events were reported.^29^

Corral-Magaña et al. evaluated 29 patients under 15 years of age with chronic urticaria, and five of them were treated with omalizumab. Four patients had a complete response to the standard dose of 300 mg administered every 4 weeks, while one patient showed a partial response with a dose of 300 mg every 2 weeks. The ages of patients ranged from 2 to 12 years, and all but one had CSU.^30^

In a real-world study spanning 16 weeks, 12 patients under 16 years of age with various types of urticaria were treated with omalizumab. Most patients (10 out of 12) received a dose of 150 mg every 4 weeks, while two 16-year-old patients received the standard dose of 300 mg every 4 weeks. Based on the UCT score, 67% of the patients achieved symptom control after the first administration. All patients with chronic inducible urticaria had their symptoms controlled within the first 3 months of treatment.^31^

A case series involving 13 children with cold urticaria (median age 13 years, range 4–16) treated with omalizumab at different doses (ranging from 75 to 450 mg every 4 weeks) was reported from eight French hospitals. Six patients experienced a complete response, six had a partial response, and the time to response was within 3 months for eight patients. No safety concerns were observed in this study.^32^

In a prospective analysis conducted by our group, 10 children and adolescents aged 8–14 years (mean age 11.4 years) with refractory CSU were treated with omalizumab at doses of 150 or 300 mg administered at different intervals (ranging from 2 to 10 weeks) based on availability and clinical response. At the time of analysis, seven patients had achieved symptom control, one had a partial response, and two did not respond to the treatment. No adverse events related to the treatment were observed.^33^

Based on our group’s findings, omalizumab treatment is recommended for children and adolescents with CSU or inducible urticaria who are refractory to a 30-day treatment with fourfold doses of a second-generation H1-antihistamine. A dose of 150 mg every 4 weeks is suggested for children under 6 years of age, while 300 mg every 4 weeks is recommended for children over 6 years and adolescents. For partial or non-responders after 3 months of treatment, an increased dose or shorter interval strategy can be considered. However, large-scale studies, especially in young children, are required to establish the efficacy and safety of omalizumab in children. Furthermore, given that the use of omalizumab in children younger than 12 years is still not part of the drug monogram; this generates an additional barrier for the use of omalizumab in young children, although it is indicated for asthmatic children over 6 years old.

### Cyclosporine and alternatives therapies in children

The identification of autoimmune antibodies to the high-affinity receptor for IgE on mast cells and IgE itself has led to trials of immunosuppressant therapy in patients with chronic urticaria. Cyclosporine (CsA), a calcineurin inhibitor, has shown effectiveness in treating CU by inhibiting activated TH cells and blocking the production of inflammatory cytokines such as IL-2, IL-3, IL-4, and TNF-alpha. IL-4 is involved in the generation of IgE, which can induce and enhance mast cell activation. CsA has been found to inhibit the IgE-mediated release of histamine from mast cells in a concentration-dependent manner and histamine secretion in patients with functional IgG antibodies against the alpha subunit of the IgE receptor.^34,35^

A meta-analysis and systematic review conducted in 2017 on the use of CsA for CU demonstrated that the overall response rate to CsA treatment with low to moderate doses at 4, 8, and 12 weeks was 54, 66, and 73%, respectively. Adverse events such as hypertension, abnormal serum creatinine, gastrointestinal symptoms, headache, hirsutism, infection, and paresthesia increased with higher doses and longer duration of CsA treatment. However, most of these events were mild and resolved after reducing the CsA dose. The recommended dosage of CsA ranges from 1 to 5 mg/kg/day, with a reasonable starting dose of 3 mg/kg/day for most patients.^36^

According to the 2022 update and revision of the EAACI/GA^2^LEN/EDF/WAO guideline, cyclosporine should be used in patients with severe CU who are refractory to four-fold doses of second-generation antihistamines and optimized doses of omalizumab treatment (up to 600 mg every 2 weeks). In such cases, CsA can be a substitute for omalizumab in doses up to 5 mg/kg/day.^1^

Several case reports and open-label studies have explored the use of CsA for CU in adults, and some reports on pediatric use have started to emerge.

A study conducted at the University of Iowa Children’s Hospital with 16 children aged 1–22 years diagnosed with antihistamine-resistant CU demonstrated the efficacy and safety of CsA in each of the sixteen children, consistent with current guideline recommendations. In this study, low-dose CsA was administered at an initial dosage of 3 mg/kg/day, twice daily. To ensure the safety of CsA, drug serum concentration was monitored prior to the morning dose when no doses were missed for at least 3 days. This monitoring facilitated safe up-dosing of CsA in children with persistent urticaria. Serum urea nitrogen and creatinine levels were monitored at regular intervals, typically every 4 weeks and more frequently after dose increases. Blood pressure measurements were taken at each clinic visit. CsA dosage was gradually reduced once urticaria was effectively suppressed for a period of 1–3 months, depending on the previous duration of CU. The reduction was done in 25 mg decrements, administered twice daily at 2-week intervals, as tolerated without a recurrence of urticaria. All patients treated with CsA experienced complete resolution of urticaria within a range of 2 days to 3 months, and no adverse events were observed in these children.^37^

Another study involving adolescents observed that CsA was effective in short-term treatment with minimum side effects after a 1-year follow-up.^38^ These studies highlight the importance of monitoring CsA adverse events at least every 4 weeks and adjusting the dosage when urticaria symptoms are controlled. Other pediatric case reports have demonstrated the efficacy of cyclosporine in CU, aiding in the tapering of chronic corticosteroid use.^39^

However, similar to adults, not all children show satisfactory disease control with the treatment options recommended by the guidelines. In cases where patients do not respond to omalizumab or when the dosage of cyclosporine is insufficient to control the severity of symptoms or due to adverse events, off-label therapies have been applied.

Staubach et al. reported two cases of children with an inadequate response to CsA (3–4 mg/kg/day) who were successfully treated with off-label dupilumab 300 mg, an anti-IL-4/IL-13 antibody approved for atopic diseases but still under trial for CU treatment. One non-responder, who had very high levels of IgE (3,528 KU/l) and atopic disease, showed inadequate response to CsA but significant improvement after 4 weeks of dupilumab treatment. The other patient took longer to show improvement with dupilumab but ultimately improved after 3 months of therapy.^40^ Improvement with dupilumab has also been reported in adult patients who did not respond to omalizumab.^41^

It is important to note that children may have a delayed response to omalizumab, as previously observed in adult studies.^42^ The decision to continue treatment with omalizumab may be based on intermittent improvement in the UAS7 and/or UCT. Staubach et al. suggest that for children with high IgE levels who do not respond to omalizumab or CsA, weight-adapted dupilumab at a dose of 200 or 300 mg every 2 weeks may be an option.^40^ Additionally, cyclosporine is not recommended for long-term treatment.

Corticosteroids are widely used for many different conditions, including urticaria. However, due to the potential long-term side effects of high doses, their use is not recommended for CSU, except for treating exacerbations for short periods (up to 10 days).^1^ Nonetheless, oral corticosteroids are still used as a third-line treatment by Southern European clinicians in 12% of children with CSU.^43^ There is currently no evidence supporting the efficacy of topical steroids in urticaria treatment.^1^

There is insufficient evidence to support the use of other immunosuppressant treatments in pediatric CU. Reports of methotrexate use are anecdotal, and its efficacy in adults with CSU is uncertain.^1,44,45^ Randomized controlled trials are necessary to formally recommend methotrexate, and at the moment, there are no data available for its use in children. Similarly, there is no evidence in children for the use of sulfasalazine, interferon, plasmapheresis, phototherapy, immunoglobulin, danazol, warfarin, tranexamic acid, hydroxychloroquine, rituximab, heparin, anakinra, anti-TNF alpha, colchicine, miltefosine, mirtazapine, or mycophenolate mofetil.^15^

While there may be an association between autoimmune diseases such as thyroiditis in children and challenging-to-control CU, there is no clear evidence that treating autoimmune thyroid disease can affect the natural course of CU. However, hormone replacement therapy is advisable in clinical practice and may have a positive effect on CU.^46^

Regarding montelukast, although it has a safety profile for atopic diseases in children, there are no specific pediatric studies on its use in CU. Moreover, this treatment step was excluded in the latest updated revisions of international guidelines since randomized trials have shown that montelukast in monotherapy does not provide better symptom improvement than antihistamines.^47^ However, although evidence from publications is limited, clinical experience suggests that montelukast may be useful in certain contexts, such as delayed pressure urticaria and non-steroidal anti-inflammatory drugs that exacerbate CU.^1^

Children should not be placed on a pseudo-allergen and additive-free diet when there is no previous and well-defined history suggesting its necessity. Studies on the efficacy of such diets have not provided evidence of their effectiveness without a clear history of adverse reactions.^1,15^

## CONCLUSIONS

CU in children significantly affects the quality of life for both the patients and their families. Therefore, an accurate diagnosis and effective treatment are crucial. The current international guidelines recommend using the same treatment algorithm for adults and children with CU. However, there are limited data available on the use of high-dose H1-antihistamines, omalizumab, and cyclosporine, which are suggested as the first, second, and third steps of treatment, respectively.^1^ Nonetheless, the existing data indicate that these drugs are safe and effective in the pediatric population when the correct dose adjustments for age and weight are made. Collaborative studies, such as those proposed by the UCARE network, will be necessary to generate stronger evidence supporting the guideline recommendations and to increase the confidence of pediatricians and specialists in treating these patients.^48^

## Data Availability

The database that originated the article is available with the corresponding author.
